# Effectiveness of cognitive behavior therapy on occupational stress management among administrative, language, science and vocational education staff within open and distance learning centers: A randomized controlled trial evaluation

**DOI:** 10.1097/MD.0000000000037231

**Published:** 2024-03-01

**Authors:** Justina Ngozi Igwe, Edith Chika Edikpa, Obodo Abigail Chikaodinaka, Mercy Ifunanya Ani, David Onyeamaechi Ekeh, Nneka Justina Eze, Bernardine Ngozi Nweze, Ifeoma Clementina Metu, Njideka Gertrude Mbelede, Chuks Marcel Ezemoyih, Christian Sunday Ugwuanyi

**Affiliations:** a Department of Adult Education and Extra-Mural Studies, University of Nigeria, Nsukka, Nigeria; b Department of Educational Foundations, University of Nigeria, Nsukka, Nigeria; c Department of Science Education, Enugu State University of Science and Technology, Enugu, Nigeria; d Department of Educational Foundations, Alex Ekwueme Federal University, Ndufu-Alike, Nigeria; e Department of Educational Management, Alex Ekwueme Federal University, Ndufu-Alike, Ikwo, Nigeria; f Department of Linguistics, Foreign and Nigerian Languages, National Open University of Nigeria, Abuja, Nigeria; g Department of Educational Foundations, Nnamdi Azikiwe University, Awka, Nigeria; h Department of Vocational and Technical Education, Alex Ekwueme Federal University, Ndufu-Alike, Nigeria; i Department of Science Education, University of Nigeria, Nsukka, Nigeria; j Department of Education Foundations, University of the Free State, Bloemfontein, South Africa.

**Keywords:** administrative, cognitive behavior therapy, distance learning centers, impact management, language, science and vocational education staff, work stress

## Abstract

**Background:**

The administrative, language, science and vocational staff in Nigerian open and distance learning centers handle a wide range of responsibilities, including teaching, supervising exams, managing projects for distant learners, conducting research, and attending conferences. However, no research in southeast Nigeria has looked into how the administrative, language, science and vocational staff at open-distance learning centers manage occupational stress. Therefore, the purpose of this study was to investigate how administrative, language, science and vocational education staff at open distance learning facilities in southeast Nigeria manage their work-related stress in relation to cognitive behavior therapy intervention.

**Methods:**

The study used a randomized control group trial design with 63 administrative, language, science and vocational staff members as the sample size. Data were gathered using the Occupational Stress Index and the Perceived Stress Scale. The instruments’ respective internal consistency reliability indices are.87 and.77. The 12-week intervention of cognitive behavior therapy was conducted. A postintervention exam was given to participants in both the intervention group and the nonintervention group after the conclusion of the intervention, and a follow-up assessment was given 2 months later. The paired samples t-test and the independent samples t-test were used to evaluate the data for the within-groups and between-groups effects, respectively.

**Results:**

In open and distance learning facilities in southeast Nigeria, it was discovered that cognitive behavior therapy significantly improved the administrative, language, science and vocational education staff’s ability to manage occupational stress.

**Conclusion:**

Administrative, language, science and vocational education staff at open distance learning facilities in southeast Nigeria can effectively manage their occupational stress through the use of cognitive behavior therapy.

## 1. Introduction

Over the past 20 years, the academic environment has evolved dramatically,^[1]^ which has led to academics experiencing occupational stress.^[2]^ Academic occupational stress has been linked to a number of negative outcomes, including decreased employee engagement, poor interpersonal connections, poor psychological well-being, poor work performance, illness, and low levels of organizational commitment.^[3,4]^ According to research, academics in Australia and New Zealand,^[5]^ Canada,^[6]^ the UK,^[2,7]^ South Africa (Dhanpat, Braine & Geldenhuys as mentioned in^[8,9]^), and Nigeria^[10–12]^ may experience significant levels of occupational stress.

In keeping with the submission above,^[13]^ discovered that Nigeria’s workplaces are overly stressful. According to,^[14]^ the main factor contributing to employees’ stress is their dangerous and threatening working conditions. Academics in open-distance learning institutions mainly rely on technology, according to,^[8]^ to stay employable and respond to students’ needs around-the-clock. As a result, one of the most frequently mentioned stresses in remote learning is the endeavor to stay current with information technology.^[9]^ Academics who are learning remotely must be reachable by phone and email around-the-clock.^[8]^ Additionally, academics who teach open-distance learning (ODL) face particular difficulties such the dynamic nature of the technology applications that support autonomous learning, flexible learning, and the development of technological communities.^[15]^

Workers of America, referenced in,^[16]^ claims that occupational stress causes low output, absenteeism, and a rise in both on- and off-the-job mishaps. According to Ekpenyong and Inyang^[17]^ and stated in,^[16]^ 39.25% of a group of Nigerian workers suffer from problems related to their line of work. Additionally,^[18]^ demonstrated that 10% of respondents in Nigeria had high work stress. It is thought that, with the right exposure to counseling therapy, such as cognitive behavior therapy (CBT), the degree of work-related stress experienced by Nigerian employees, particularly those employed by open distance learning institutions, can be controlled.

A psychological intervention based on emotions, cognition, and scientific theories of human behavior is called CBT.^[19]^ By altering beliefs and actions, CBT is a collection of psychotherapy methods that de-emphasizes illogical behavior.^[20]^ According to Zafer,^[21]^ CBT seeks to lower patients’ stress levels, address the symptoms of the disease, reassess patients’ cognitive capacities, and encourage positive behavior changes. James et al^[22]^ state that many mental diseases can be managed using various therapeutic modalities. During therapy sessions, patients and therapists collaborate to comprehend and recognize the obstacles related to behavior, emotions, and thoughts being in sync.^[23]^ Numerous studies have demonstrated the efficacy of cognitive behavior therapy (CBT) in helping university students and employees manage their work-related stress or any type of illogical thinking.

According to Sarida, Bergerb, and Segal-Engelchina,^[24]^ CBI significantly affects nurses’ mood states, perceived stress, and sense of coherence (SOC). Rational-emotive behavior therapy’s cognitive restructuring intervention program dramatically decreased irrational thinking resulting from traumatic childhood experiences.^[25]^ Modified cognitive behavioral therapy (CBT) was shown to reduce anxiety, depression, and obsessive-compulsive disorder (OCD) (Sasha, Maria, and Ailsa).^[26]^ In patients with work-related stress complaints, Dalgaard et al^[27]^ discovered a significant impact of a stress-management intervention (CBT) on a long-lasting return to work. Comparing the Work-focused Cognitive Behavioral Intervention group to the control group, Dalgaard et al^[28]^ discovered substantial group effects on felt stress and memory. Following their exposure to rational emotive cognitive-behavioral coaching (RE-CBC), individuals in the group showed a significant drop in depression, according to Eseadi et al^[29]^ Zafer^[21]^ discovered that the number of people with mental diseases is declining, indicating that cognitive behavioral treatment (CBT) is a modern, recognized, and successful way of treating patients with illogical thinking. According to Lauren and Kate,^[30]^ clients who participated in the CBT intervention program reported feeling less angry and more confident in themselves. According to Onuigbo et al,^[31]^ students who received rational emotive behavior therapy significantly reduced their depression levels at follow-up and posttreatment evaluations; the control group did not see any similar changes. According to Ugwuanyi et al,^[32]^ individuals who were a part of the CBT-music intervention program exhibited notably reduced test anxiety ratings at the posttreatment phase in comparison to those in the control group. According to Ugwuanyi et al,^[33]^ college students studying physics, chemistry, and mathematics who procrastinate on their assignments find that cognitive behavior therapy significantly reduces this tendency. The aforementioned empirical data makes it clear that cognitive behavioral therapy (CBT) is useful in helping students and employees manage their work-related stress and other related illogical beliefs. The effect of CBT on managing occupational stress in administrative workers at open distance learning institutions in Southeast Nigeria, however, was not taken into account in any of the investigations.

The current study was required due to the aforementioned factors as well as the paucity of literature on the effects of cognitive behavioral therapy (CBT) on the management of occupational stress among administrative, language, science and vocational staff in open distance learning centers in the Southeast States of Nigeria. Thus, the research question posed for this research was: What is the effectiveness of cognitive behavior therapy on occupational stress management among administrative, language, science and vocational staff within open and distance learning centers? The researchers therefore postulated that CBT had no appreciable effect on the administrative, language, science and vocational education staff’s management of occupational stress in open distance learning centers.

## 2. Methods

### 2.1. Design of the study

The experimental design used was a randomized control trial with a pretest and posttest. Randomization was used to assign subjects to intervention or nonintervention groups. Recent studies like^[34–38]^ have adopted this kind of design in similar experimental conditions.

### 2.2. Participants

**Figure 1.**
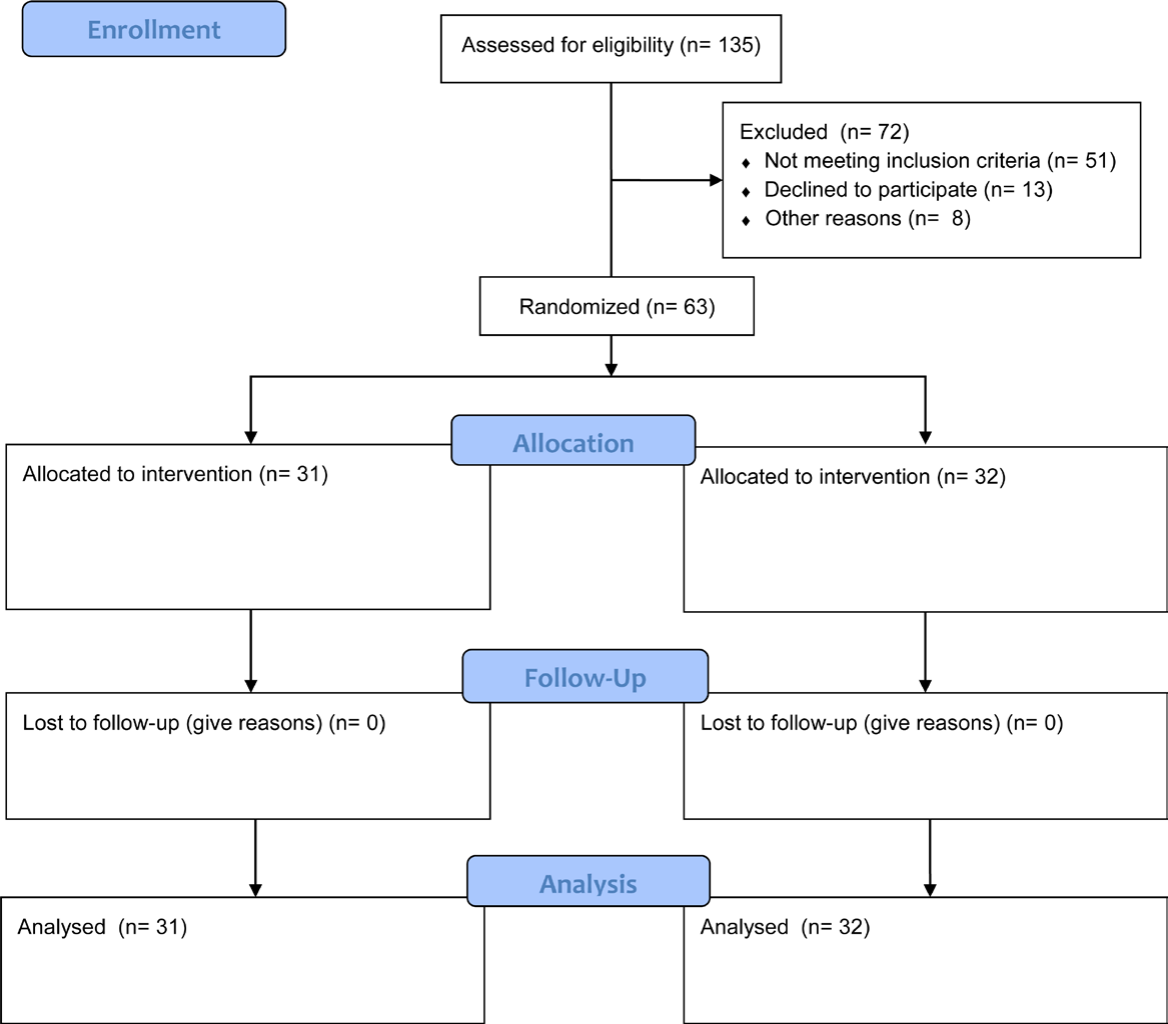
Flow diagram of the participants.

A total of 63 administrative, language, science and vocational education staff who were randomly sampled from all the ODL centers in South East (SE), Nigeria constituted the sample size for the study. This number was gotten from the 135 administrative, language, science and vocational staff who volunteered to participate in the intervention program at the conclusion of the advertisement period. The administrative, language, science and vocational staff in those centers were recruited through their WhatsApp platforms. Since participation in the intervention program was voluntary, the participants were asked to indicate their interest in participating in the CBT intervention program. The following eligibility criteria were used to screen these participants: (1). Must be employed by any ODL centers in Southeast Nigeria as either administrative, language, science or vocational education staff. (2). must have stress-related symptoms following the occupational stress index baseline assessment (OSI). (3). Has to be engaged in WhatsApp conversations. Following the eligibility check, 63 individuals were chosen in accordance with the eligibility requirements to become study participants. G-Power, version 3.1 yielded a sample size of.82, which is sufficient for this investigation.^[39]^ Randomization was used to assign the individuals to 31 intervention and 32 nonintervention groups as shown in Figure 1.

### 2.3. Measures

The study used the Occupational Stress Index, which was created by Srivastava and Singh in 1984. The OSI is a 46-item measure that evaluates how much stress workers endure in relation to their overall well-being. For OSI, there are 5 possible answers: 5 for absolutely true, 4 for almost true, 3 for mostly true, 2 for nearly false, and 1 for totally false. We total the scores on each statement to get an estimate of the amount of occupational stress experienced by workers. A worker is considered to be under stress at work if their score is <115, moderately stressed if it is between 116 and 161, and extremely stressed if it is >161. The OSI items have an internal consistency dependability of.87.

For the study, the Perceived Stress Scale created by Cohen et al (1983) was used. The perceived stress scale (PSS) is a self-reported, unidimensional, 10-item test designed to gauge an individual’s perceived stress in relation to various life circumstances. The most used psychological tool for gauging stress perception is the Perceived Stress Scale. It is a gauge of how stressful one feels about certain circumstances in their life. The purpose of the items was to gauge how erratic, unmanageable, and hectic the lives of the respondents were. A 5-point rating system was used to organize the PSS items: Never (0), Almost Never (1), Sometimes (2), Fairly Often (3), and Very Often (4). The lowest possible score is 0; the greatest possible score is 40. These questions probe the worker’s emotions, ideas, and behaviors regarding his or her living and working settings. Among these are the following: how often have you been disturbed due to an unforeseen event in the past month? The PSS items have an internal consistency reliability of 0.77.

### 2.4. Procedure

Before the intervention program started, the administrative personnel at those locations advertised the program and asked them to express their interest in participating through different WhatsApp channels. 135 administrative employees expressed interest in taking part in the program through that channel. Subsequently, the researchers proceeded to conduct the OSI on program volunteers in order to determine their eligibility according to the established eligibility requirements. 63 individuals who satisfy the inclusion or eligibility requirements are the outcome of the selection process. At that moment, the chosen subjects were given the PSS in order to obtain the study’s second baseline data.

Next, groups for the intervention and nonintervention were randomly assigned to the individuals. The goals of the study and the methodology for implementing the program were well explained to both groups. Zoom offered both the 12-week intervention and nonintervention programs. Zoom uses a peer-to-peer cloud-based software platform to offer online chat and video telephone services. The initial Zoom online meeting served as a means of familiarization and creating a supportive environment for the program’s implementation. An agreement was reached to give participants data bundles as a way to keep them motivated and to guarantee their active engagement in the program. The meeting day and time were decided upon as follows: Tuesday and Thursday from 5 to 7 pm for an average period of 12 weeks, commencing on May 11, 2023, and ending on July 10, 2023. Participants in the nonintervention group received standard conventional therapy throughout this time, while those in the intervention group were exposed to the CBT intervention program. The PSS was given to the participants at the conclusion of the program in order to gather posttest data.

A follow-up measure was collected using the OSI and PSS 2 months following the intervention program (September 19, 2023) to determine the participants’ level of retention of the influence of the CBT. Throughout the recruitment, treatment, and data analysis phases, blinding was maintained for research assistants, participants, and data analysts. After being cleaned, the data from the pretest, posttest, and follow-up measurements were analyzed.

### 2.5. CBT intervention program

For this investigation, the CBT intervention program handbook for people with irrational thinking was modified from.^[40]^ Muñoz et al^[40]^ state that the foundation of cognitive-behavioral therapy is the link between ideas, behaviors, and emotions. This approach emphasizes the significance of recognizing the ideas and behaviors that impact the work experience in order to help adolescents manage their stress levels. This will help them learn to take charge of their emotions. Therapy sessions are broken down into 3 modules, each of which has 4 sessions, in this manual.

#### 2.5.1. Sessions 1–4.

Information regarding how participants’ thoughts affect their work experience is presented in these sessions. This module’s first session sets the tone and goals for the rest of the sessions. In addition, the parameters for the therapy, the day and time of the sessions, and the boundaries of secrecy were appropriately set. This module aims to educate participants on the boundaries and extent of secrecy, as it has the potential to impact the nature and caliber of therapeutic alliances.

A discussion of occupational stress – what it is and how individuals experience it – opened the first session. The therapist also explained during this session the goal of the first module, which is to define thoughts precisely in order to help participants understand how they affect their job experience. The topics of the following 3 sessions were various forms of cognitive mistakes and dysfunctional thought patterns related to work-related stress. Additionally, the participants were made aware of how to discuss and alter these dysfunctional thoughts and thinking errors linked to occupational stress in order to manage occupational stress. Some tasks are designed to help discover thinking flaws in between sessions. Additionally, throughout the sessions, the participants were introduced to techniques for reducing unhealthy or dysfunctional negative thoughts and raising good ones, which in turn helped to lessen symptoms of occupational stress.

#### 2.5.2. Sessions 5–8.

The participants were able to link symptoms of work stress and enjoyment of activities during sessions 5 to 8. There was a talk about how work-related stress might make it harder to engage in enjoyable activities, which exacerbates stress-related illnesses. In these sessions, enjoyable activities were described and barriers to participation were noted. Additionally, the participants were given opportunities to set specific goals, which can lessen work-related stress. The participants were coached on how to define realistic goals, and those actions were practiced during the designated coaching sessions. Sessions 5 through eighteen centered on giving the participants more power over their life and teaching them how to recognize options that will provide them greater autonomy and choice. The therapist assisted the participants in creating realistic goals and engaging in activities that enhanced their job experiences.

#### 2.5.3. Sessions 9–12.

These sessions gave the participants an overview of how their relationships impact their work experience by outlining the benefits of social support and how it may be useful in handling challenging circumstances. The participants were able to discover and fortify their social support networks through these sessions. The final sessions bring together the themes covered in the earlier modules. Together with the participants, the therapist looked at how the participants’ thoughts influenced their relationships, social support system, and activities. Assertive communication techniques were taught to the participants through exercises, which would aid in the establishment of fulfilling and healthy relationships. The major topics of each module were reexamined and integrated as the CBT intervention program came to a close. In order to pinpoint the participants’ strengths and accomplishments, an assessment of the treatment experience was conducted with them during the last session.

### 2.6. Data analysis

**Figure 2.**
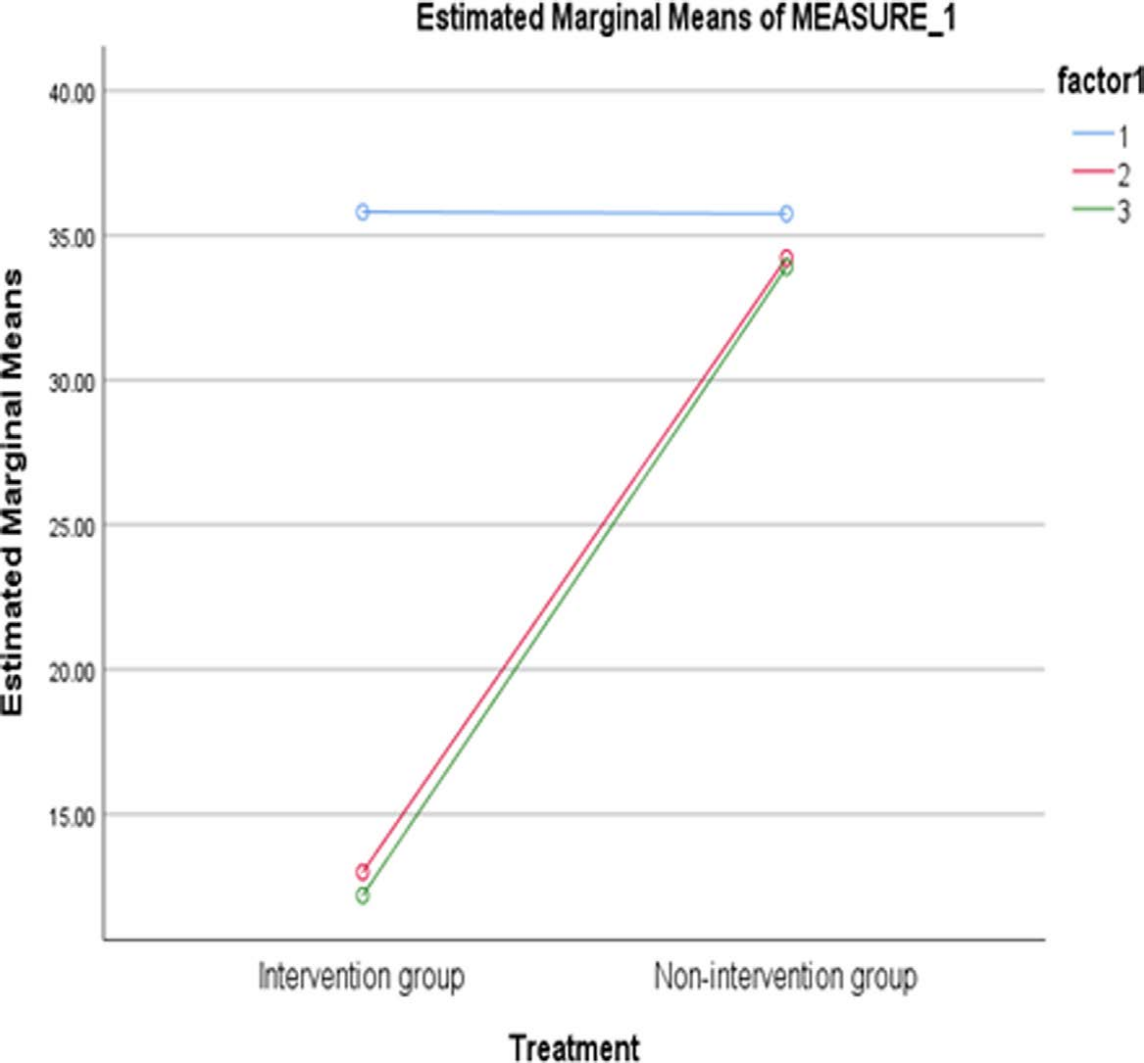
Interaction graph of time and treatment.

The statistical analysis was done using SPSS software, version 25. The data was statistically analyzed using the mean, the *t* test of independent samples, and the *t* test of pairs of samples. The hypotheses were tested using a 2-tailed test. Partial Eta squared (ŋ^2^) value was used to report the intervention’s effect size on the administrative, language, science and vocational education staff’s management of occupational stress in ODL centers. The corresponding author is in possession of the study’s data, which can be accessed upon request. Figure 2 displays a summary of the materials and procedures.

### 2.7. Ethical approval

The conduct of this study was approved by the Faculty of Education Ethical Committee on Research at the University of Nigeria, Nsukka, Nigeria with committee approval number REC/FoE/2023/000113. The Declaration of Helsinki, which outlines moral guidelines for medical research involving human people, was adhered to by the researchers. Prior to the start of the treatment, the participants were also given informed consent papers to sign.

## 3. Results

Table 1 shows that there was a significant difference in the proportion of male and female administrative, language, science and vocational staff participants in the study in favor of the male participants (χ^2^(1) = 12.65, *P* = .05). Besides, there were significant differences in the participants’ age, tribe and religion: χ^2^(2) = 9.43, *P* < .050; χ^2^(2) = 34.54, *P* < .050; χ^2^(2) = 41.56, *P* < .050.

**Table 1:** Participants’ demographic profiles.

Demographics		Intervention group n (%)	Control group n (%)	χ^2^	*P*
Gender	Male	19 (61.29)	22 (68.75)		
	Female	12 (38.71)	11 (34.38)	12.65	<.05
Age	20–25	4 (12.90)	5 (15.63)	9.43	<.05
	26–35	12 (38.71)	15 (46.88)		
	≥36	15 (48.39)	12 (37.50)		
Tribe	Igbo	23 (74.19)	23 (71.88)	34.54	<.05
	Yoruba	5 (16.13)	7 (21.88)		
	Housa	3 (9.68)	2 (6.25)		
Religion	Christian	27 (87.10)	27 (84.38)	41.56	<.05
	Moslem	4 (12.90)	5 (15.63)		

Participants in the intervention group’s perceived stress rating (M = 35.85, SD = 1.68), as measured by PSS, was almost identical to those in the nonintervention group (M = 35.81, SD = 1.66) at the pretest (see Table 2). A posttest analysis revealed, however, that the nonintervention group’s perceived stress level (M = 34.21, SD = 4.52) was higher than the intervention group’s (M = 13.34, SD = 2.26). During the follow-up, the intervention group’s mean perception of stress (M = 12.19, SD = 2.27) was lower than the nonintervention group (M = 33.90, SD = 4.57).

**Table 2:** Mean analysis of the occupational stress ratings of the participants.

Treatment	n	Pretest (1)		Posttest (2)		Follow-up (3)	
		Mean	SD	Mean	SD	Mean	SD
Intervention	31	35.85	1.68	13.34	2.26	12.19	2.27
Control	32	35.75	1.68	34.21	4.52	33.90	4.57

The management of occupational stress among administrators of ODL centers was significantly different across the 3-time measures, *F* (2, 122) = 650.940, *P* < .050, ŋ^2^ = .914, and between groups, *F* (1, 61) = 7534.331, *P* < .050, ŋ^2^ = .992 (see Table 3). Also, time and treatment exhibited a significant interaction, *F* (2, 122) = 485.878, *P* < .050, ŋ^2^ = .532 (see Fig. 2). All measure pairs exhibit mean differences that are significant at *P* < .050, according to Table 4. This indicates that the mean differences between measures 2 and 3, 3 and 2, did not affect the significant effect of time on the participants’ work stress.

**Table 3:** Within-subjects effect and between-subjects effects of the intervention using Mixed design repeated measures.

Measure	Source		Type III sum of squares	df	Mean square	*F*	Sig.	Partial eta squared
Tests of within-subjects effect								
PSS	Time	Sphericity Assumed	6516.847	2	3258.423	650.940	.000	0.914
	Time * Treatment	Sphericity Assumed	4864.339	2	2432.169	485.878	.000	0.888
	Error (Time)	Sphericity Assumed	610.698	122	5.006			
Tests of between-subjects effect								
PSS	Intercept		142,678.812	1	142,678.812	7534.331	.000	0.992
	Treatment		9648.463	1	9648.463	509.499	.000	0.893
	Error		1155.167	61	18.937			

ŋ^2^ = effect size.

**Table 4:** Pairwise comparisons.

(I) factor1	(J) factor1	Mean difference (I-J)	Std. error	Sig.†	95% confidence interval for difference†	
					Lower bound	Upper bound
1	2	12.169*	0.459	0.000	11.038	13.300
	3	12.728*	0.478	0.000	11.550	13.906
2	1	−12.169*	0.459	0.000	−13.300	−11.038
	3	0.559*	0.192	0.015	0.087	1.032
3	1	−12.728*	0.478	0.000	−13.906	−11.550
	2	−0.559*	0.192	0.015	−1.032	−0.087

Based on estimated marginal means.

* The mean difference is significant at the 0.05 level.

† Adjustment for multiple comparisons: Bonferroni.

The ability of the nonintervention group to handle occupational-related stress did not vary over time, according to a subsequent examination of this interaction, and there was no statistically significant difference between the baseline. However, the mean occupational stress ratings of the intervention group steadily decreased, indicating that CBT intervention had a significant effect on the ODL administrators’ capacity to manage occupational stress.

## 4. Discussion

This study looked at how cognitive behavioral therapy (CBT) affected the administrative, language, science and vocational education staff of ODL centers in southeast Nigeria that treat occupational stress. The study’s conclusions demonstrated that the CBT intervention program significantly improved the administrative, language, science and vocational staff of ODL centers’ ability to manage occupational stress. These results help to understand why the CBT intervention program is so engaging. This research has demonstrated that the intervention program has the innate capacity to convert clients’ illogical ideas or beliefs into reasonable ones. Comparable research utilizing other participants has shown the effect of CBT intervention program on the management of illogical ideas such as test anxiety, burnout, or stress.

According to Sarida et al,^[24]^ CBI significantly affects nurses’ mood states, perceived stress, and sense of coherence (SOC). According to Eseadi et al,^[25]^ the rational-emotive behavior therapy cognitive restructuring intervention program dramatically decreased irrational beliefs resulting from traumatic childhood stressors. Modified cognitive behavioral therapy (CBT) was shown to reduce anxiety, depression, and obsessive-compulsive disorder (OCD) (Sasha, Maria, and Ailsa,.^[26]^ In patients with work-related stress complaints, Dalgaard et al^[27]^ discovered a significant impact of a stress-management intervention (CBT) on a long-lasting return to work. Comparing the Work-focused Cognitive Behavioral Intervention group to the control group, Dalgaard et al^[28]^ discovered substantial group effects on felt stress and memory. Following their exposure to rational emotive cognitive behavioral coaching (RE-CBC), individuals in the group showed a significant drop in depression, according to Eseadi et al^[29]^ Zafer^[21]^ discovered that the number of people with mental diseases is declining, indicating that cognitive behavioral treatment (CBT) is a modern, recognized, and successful way of treating patients with illogical thinking. According to Lauren and Kate,^[31]^ clients who participated in the CBT intervention program reported feeling less angry and more confident in themselves. Following treatment, test anxiety levels were considerably lower for individuals in the CBT-music intervention program than for those in the control group.^[32]^ Undergraduate students studying physics, chemistry, and mathematics reported a significant reduction in academic procrastination after receiving cognitive behavior therapy.^[33]^

## 5. Limitations of the study

The bad browsing network that was experienced during the intervention program may have limited the study’s conclusions. Due to a bad browsing network, there were barriers in the way of the intervention contents during the intervention period. Although the intervention technique was designed to flow well, care should be used when interpreting the findings’ generalizability. Furthermore, the impact of CBT on occupational stress could not be examined by the researchers in relation to relevant modifiers like gender, age, religion, or tribe. Therefore, based on this, the researchers recommended that future researchers replicate the study by meeting in person and taking into account any potential moderating influence on the impact of CBT on administrative, language, science and vocational education staff members’ occupational stress.

## 6. Conclusion and strength of the study

According to this study, cognitive behavioral therapy (CBT) is a very useful tool for managing occupational stress in administrative, language, science and vocational education workers in ODL centers in South-East Nigeria. This has added to the body of knowledge already available in the field of science education since it is the first study to demonstrate the efficacy of cognitive behavioral therapy (CBT) in managing occupational stress in administrative, language, science and vocational staff of ODL centers located in the southeast of Nigeria. There was no empirical data on the topic prior to the publication of this study output. This finding has implications for community development because it shows that when ODL centers’ administrative, language, science and vocational staff can effectively manage their work-related stress, they will do their best to prepare students there, which will enable students to contribute to the development of the communities to which they belong.

Based on the study’s findings, the researchers advise that the appropriate ODL authorities host seminars and workshops where administrative, language, science and vocational staff members can receive CBT intervention program counseling. Periodically, this lecture or workshop should be held to help the administrative personnel manage the demands of the ODL workload.

## Acknowledgments

The directors of the sampled ODL facilities cooperated with the researchers, and they are grateful to all of the study participants.

## Author contributions

**Conceptualization:** Justina Ngozi Igwe, Edith Chika Edikpa, Obodo Abigail Chikaodinaka, Mercy Ifunanya Ani, David Onyeamaechi Ekeh, Nneka Justina Eze, Bernardine Ngozi Nweze, Njideka Gertrude Mbelede, Chuks Marcel Ezemoyih.

**Funding acquisition:** Justina Ngozi Igwe, Edith Chika Edikpa, Obodo Abigail Chikaodinaka, Mercy Ifunanya Ani, David Onyeamaechi Ekeh, Nneka Justina Eze, Bernardine Ngozi Nweze, Ifeoma Clementina Metu, Njideka Gertrude Mbelede, Chuks Marcel Ezemoyih.

**Investigation:** Justina Ngozi Igwe, Edith Chika Edikpa, Obodo Abigail Chikaodinaka, Mercy Ifunanya Ani, David Onyeamaechi Ekeh, Nneka Justina Eze, Bernardine Ngozi Nweze, Ifeoma Clementina Metu, Njideka Gertrude Mbelede, Chuks Marcel Ezemoyih.

**Methodology:** Justina Ngozi Igwe, Edith Chika Edikpa, Obodo Abigail Chikaodinaka, Mercy Ifunanya Ani, David Onyeamaechi Ekeh, Nneka Justina Eze, Bernardine Ngozi Nweze, Njideka Gertrude Mbelede.

**Project administration:** Justina Ngozi Igwe, Edith Chika Edikpa, Obodo Abigail Chikaodinaka, Mercy Ifunanya Ani, David Onyeamaechi Ekeh, Nneka Justina Eze, Bernardine Ngozi Nweze, Ifeoma Clementina Metu, Njideka Gertrude Mbelede, Chuks Marcel Ezemoyih.

**Resources:** Justina Ngozi Igwe, Edith Chika Edikpa, Obodo Abigail Chikaodinaka, Mercy Ifunanya Ani, David Onyeamaechi Ekeh, Nneka Justina Eze, Bernardine Ngozi Nweze, Ifeoma Clementina Metu, Njideka Gertrude Mbelede, Chuks Marcel Ezemoyih.

**Supervision:** Justina Ngozi Igwe, Edith Chika Edikpa, Chuks Marcel Ezemoyih.

**Validation:** Mercy Ifunanya Ani.

**Visualization:** Justina Ngozi Igwe, Edith Chika Edikpa.

**Writing – original draft:** Justina Ngozi Igwe, Edith Chika Edikpa.

**Writing – review & editing:** Justina Ngozi Igwe, Edith Chika Edikpa.

**Data curation:** Christian Sunday Ugwuanyi.

**Formal analysis:** Christian Sunday Ugwuanyi.

**Software:** Christian Sunday Ugwuanyi.
